# Association of *Nrf2*, *SOD2* and *GPX1* Polymorphisms with Biomarkers of Oxidative Distress and Survival in End-Stage Renal Disease Patients

**DOI:** 10.3390/toxins11070431

**Published:** 2019-07-23

**Authors:** Djurdja Jerotic, Marija Matic, Sonja Suvakov, Katarina Vucicevic, Tatjana Damjanovic, Ana Savic-Radojevic, Marija Pljesa-Ercegovac, Vesna Coric, Aleksandra Stefanovic, Jasmina Ivanisevic, Zorana Jelic-Ivanovic, Lana McClements, Nada Dimkovic, Tatjana Simic

**Affiliations:** 1 Institute of Medical and Clinical Biochemistry, Faculty of Medicine, University of Belgrade, 11000 Belgrade, Serbia; 2 Faculty of Medicine, University of Belgrade, 11000 Belgrade, Serbia; 3 Department of Pharmacokinetics and Clinical Pharmacy, Faculty of Pharmacy, University of Belgrade, 11000 Belgrade, Serbia; 4 Clinical Department for Renal Diseases, Zvezdara University Medical Center, 11000 Belgrade, Serbia; 5 Department of Medical Biochemistry, Faculty of Pharmacy, University of Belgrade, 11000 Belgrade, Serbia; 6 School of Life Sciences, Faculty of Science, University of Technology Sydney, NSW 2007 Sidney, Australia; 7 Serbian Academy of Sciences and Arts, 11000 Belgrade, Serbia

**Keywords:** end-stage renal disease, hemodialysis, survival, Nrf2, SOD2, GPX1, polymorphism, oxidative stress

## Abstract

The oxidative stress response via Nuclear factor (erythroid-derived 2)-like 2 (Nrf2) interlinks inflammation- and metabolism-related pathways in chronic kidney disease. We assessed the association between polymorphisms in Nrf2, superoxide dismutase (SOD2), glutathione peroxidase (GPX1), and the risk of end-stage renal disease (ESRD). The modifying effect of these polymorphisms on both oxidative phenotype and ESRD prognosis, both independently and/or in combination with the glutathione S-transferase M1 (*GSTM1*) deletion polymorphism, was further analyzed. Polymorphisms in *Nrf2* (rs6721961), *SOD2* (rs4880), *GPX1* (rs1050450), and *GSTM1* were determined by PCR in 256 ESRD patients undergoing hemodialysis and 374 controls. Byproducts of oxidative stress were analyzed spectrophotometically or by ELISA. Time-to-event modeling was performed to evaluate overall survival and cardiovascular survival. The *SOD2 Val*/*Val* genotype increased ESRD risk (OR = 2.01, *p* = 0.002), which was even higher in combination with the *GPX1 Leu/Leu* genotype (OR = 3.27, *p* = 0.019). Polymorphism in *SOD2* also showed an effect on oxidative phenotypes. Overall survival in ESRD patients was dependent on a combination of the *Nrf2* (*C*/*C*) and *GPX1 (Leu*/*Leu)* genotypes in addition to a patients’ age and *GSTM1* polymorphism. Similarly, the *GPX1 (Leu*/*Leu)* genotype contributed to longer cardiovascular survival. Conclusions: Our results show that *SOD2*, *GPX1*, and *Nrf2* polymorphisms are associated with ESRD development and can predict survival.

## 1. Introduction

In addition to being well recognized, distinct causes of chronic kidney disease (CKD) such as diabetes mellitus, hypertension, glomerulonephritis and hereditary diseases, the genetic characteristics of CKD patients have also been identified as key factors involved in the development and prognosis of the disease. Genome-wide association studies (GWAS) identified several genetic loci with highly significant associations with CKD [1]. Transcriptomic data for candidate genes identified in these studies, were further validated in the European Renal cDNA Bank-Kroener-Fresenius Biopsy Bank (ERCB) and C-PROBE cohorts [2,3]. This systemic biology approach revealed that the pathways associated with CKD were aggregated into a network of two main clusters comprising inflammation- and metabolism-related pathways, with the nuclear factor erythroid 2-related factor 2 (Nrf2)-mediated oxidative stress response pathway serving as the hub between these two clusters [3]. The Nrf2 transcription factor is effective in promoting expression of genes that have one or more antioxidant response elements (ARE) or electrophile response elements (EpRE) in their promoter regions [4]. Nrf2 is negatively regulated through protein–protein interactions with Kelch-like ECH-associated protein 1 (Keap1). Specifically, Nrf2 is present in the cytoplasm as an inactive complex bound to Keap1 that facilitates Nrf2 ubiquitination, followed by proteasomal degradation [5]. The Nrf2–Keap1 complex serves as a redox sensor responsible for the cellular response to electrophiles. This function enables the long-term induction of protective anti-oxidant Phase II enzyme systems in a chronic disease setting [6].

The polymorphisms in *Nrf2*, superoxide dismutase (*SOD2*), and glutathione peroxidase (*GPX1*) genes, individually, have been implicated previously in disease susceptibility or prognosis in end-stage renal disease (ESRD), CKD, or diabetic nephropathy patients [7,8,9,10,11]. While Nrf2 is the master regulator of redox-mediated proteins expression, GPX1 and SOD2 have complementary functions as key enzymatic factors abrogating the level of superoxide and hydrogen peroxide, abundantly produced in conditions characterized by uraemia and inflammation, such as ESRD [12]. An interesting approach to elucidate the mechanisms, by which genetic variations influence ESRD development and its prognosis, was applied in a study investigating the role of glutathione S-transferase (GST) family members, which are known as Nrf2 targets. *GST* gene variations, particularly the deletion polymorphism of the *GSTM1* gene, have been shown to influence not only the risk of ESRD development, but also oxidative and clinical phenotypes in ESRD patients [13,14]. In light of the presented evidence, we hypothesized that polymorphisms in regulatory and catalytic proteins of antioxidant defense, such as Nrf2, SOD2, and GPX1, individually and in combination, have a direct effect on the level of oxidative stress and, consequently, impact negatively on the prognosis of ESRD patients undergoing hemodialysis.

The aim of our study was to assess the association between *Nrf2* rs6721961, *SOD2* rs4880, and *GPX1* rs1050450 polymorphisms, individually and in combination, with the risk of ESRD development and their role in the production of oxidative stress byproducts in ESRD patients on hemodialysis. In addition, we investigated the predictive value of *Nrf2*, *SOD2*, and *GPX1* polymorphisms, in the presence or absence of the *GSTM1* polymorphism, on the survival of ESRD patients on hemodialysis.

## 2. Results

Demographic and clinical characteristics of 630 study subjects (256 patients with ESRD and 374 controls) are presented in Table 1. Participants did not differ significantly in terms of age or gender. As expected, differences were observed in the smoking status, presence of diabetes or hypertension, BMI, and biochemical serum parameters.

### 2.1. Positive Association Between the Presence of SOD2, GPX1 and Nrf2 Polymorphisms and the Risk of ESRD

Analyses of our samples implicated that only the *SOD2* low-activity genotype (*Val/Val*), as an individual polymorphism, is associated with the risk of ESRD development (OR = 2.01, 95%CI = 1.28–3.16, *p* = 0.002; Table 2). Although *GPX1* and *Nrf2* polymorphisms individually did not show an association with ESRD risk, individuals who carried both *SOD2* and *GPX1* low-activity genotypes (*SOD2 Val/Val* and *GPX1 Leu/Leu*) were at the highest risk of ESRD development (OR = 3.27, 95%CI = 1.12–8.25, *p* = 0.019). *SOD2 Val/Val* with a more active, *C/C* form of the *Nrf2* gene was also associated with an increased risk of ESRD development (OR = 1.8, 95%CI = 1.14–2.82, *p* = 0.011). The observed associations remained significant even after the Bonferroni correction. 

### 2.2. Association of SOD2, GPX, and Nrf2 Polymorphisms with Biomarkers of Oxidative Damage

*SOD2* polymorphism had a significant impact on the levels of protein oxidative damage byproducts. Namely, *SOD2 Val/Val* homozygotes had lower concentrations of thiol groups (PSH) when compared to *SOD2 Ala* carriers (*p =* 0.049), as well as a higher content of carbonyl groups in comparison to the *SOD2 Ala/Ala* homozygotes (*p =* 0.037). Both malondialdehyde (MDA) and MDA adducts, byproducts of lipid oxidative damage, were elevated in *SOD2 Val/Val* homozygotes compared to *Ala* carriers (*p =* 0.036 and *p =* 0.046, respectively). MDA was also elevated compared to *Ala/Ala* referent homozygotes. Similarly, total oxidant status (TOS) and the prooxidant–antioxidant balance (PAB) concentrations were increased in patients with *SOD2 Val/Val* genotypes compared to *Ala* carriers, but only the elevation of PAB reached statistical significance (*p =* 0.044). No significant associations were observed between *GPX1* and *Nrf2* polymorphisms and any of the biomarkers of oxidative damage measured (Table 3).

### 2.3. The Influence of SOD2, GPX1, Nrf2 and GSTM1 Polymorphisms on Patient Survival

The uniform hazard model was the most appropriate to use with our overall survival data, and the corresponding shape parameter (T) followed lognormal distribution. In the covariate analysis, the patients’ age was identified as a predictor of survival, with the greatest decrease in objective function value (OFV)(reduction by 31.24 units). The model of overall survival was further improved by the addition of the *GSTM1* polymorphism and a combination of *Nrf2* and *GPX1* polymorphisms as two-level categorical covariates in the model. In the following steps, the inclusion of the remaining covariates did not produce a statistically significant decrease in the OFV. In the backward steps, no covariate was excluded from the full model. Details of the model building are given in Table S1 of the Supplementary Materials. These details show that in a unicovariate analysis, the combined *SOD2* and *GPX1* polymorphisms significantly decreased the OFV value (*p* < 0.05) as the second step of covariate testing. However, its predictability of survival was less significant than the age, *GSTM1*, and *GPX1/Nrf2* combined genotype. Hence, the developed final hazard model for the overall survival function was calculated as follows:

$$h_{t}=\frac{1}{Te-t},\;\mathrm{where}\;Te=139\cdot {\left(\frac{AGE}{65}\right)}^{-2.27}\cdot \{\begin{array}{l} e^{0.765},\;if\;Nrf2\;C/C+GPX1\;Leu/Leu\\ e^{-0.357},\;if\;GSTM1\;null\;genotype \end{array}$$

The estimated parameters of the hazard function and standard errors of the corresponding parameters of the final model are presented in Table S2 of the Supplement.

**Figure 1:**
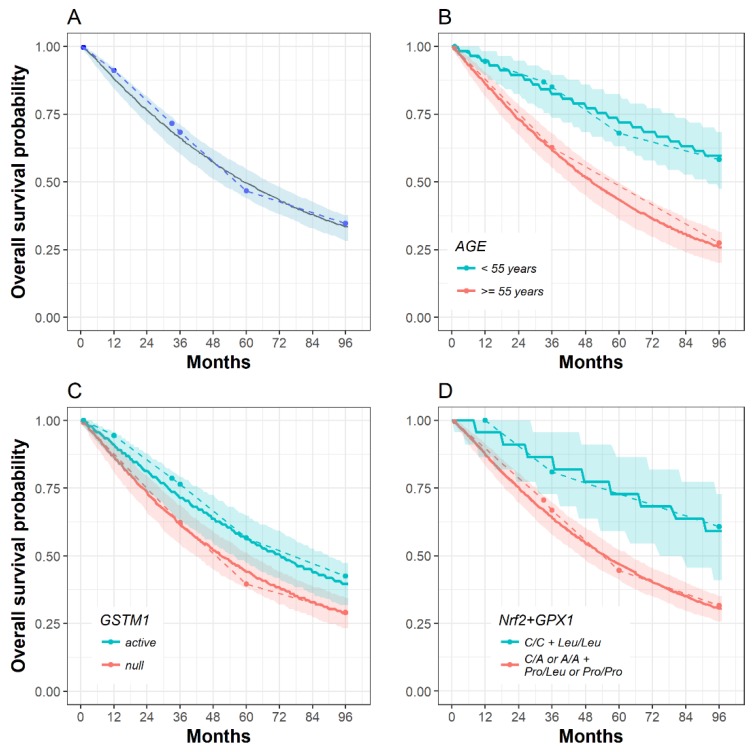
Kaplan–Meier curves for the overall survival of observed events (dashed line), including censored data (circle) and the survival model with uniform hazard estimates (solid line) and its 90% prediction interval (shaded area). No covariate stratification (**A**); stratification by final survival model covariates: age (**B**); the Glutathione S-transferase M1 ( *GSTM1)* active genotype is associated with longer overall survival (**C**); the “best survival” genotypes of the *Nrf2 (C/C*) and *GPX1 (Leu/Leu*) are associated with longer overall survival (**D**).

The empirical and model-predicted Kaplan–Meier plots of survival probability with regard to the covariates included in the survival model suggest that this model is suitable for predicting survival in our cohort of patients (Figure 1).

The greatest benefit of this model is its predictability. It can be used to analytically calculate the probabilities of survival using patients’ characteristics. It can also simulate Kaplan–Meier curves with prediction intervals based on a number of patients’ characteristics. Table 4 shows the probabilities of overall survival at three time points (3, 5, and 8 years). According to our results, the average patient aged 55, who is a carrier of the best survival genotype (*GSTM1* active *and Nrf C/C*+*GPX1 Leu/Leu*) has a much better probability of 8 yr overall survival when compared to an age-mate carrying the *GSTM1* null and *Nrf C/A* or *A/A* + *GPX1 Pro/Leu* or *Pro/Pro* genotypes (75.43% vs. 12.48%).

The empirical Kaplan-Meier plots for the *SOD2, Nrf2* and *GPX1* polymorphisms, which were not included in the model, are given in Figure S1 of the Supplementary Materials.

The time-to-event model with a Weibull hazard and the corresponding shape parameter (T) following lognormal distribution adequately described the cardiovascular survival data. After the stepwise covariate inclusion procedure, the developed cardiovascular survival model included the combined impact of a patient’s age as a continuous variable and the polymorphisms of *GSTM1* and *GPX1* as two-level categorical variables. As expected, the patients’ ages had the greatest prognostic influence on survival (decreasing OFV by 18.28 units; data not shown). Furthermore, model improvement was achieved by the addition of *GSTM1* and *GPX1* polymorphisms that, in total, reduced OFV for an additional 12.11 units (Data not shown). In the backward steps, no covariate was excluded from the full model. A summary of the covariate testing is given in Table S3 of the Supplementary Materials. This summary shows that when polymorphism variables were tested against the hazard model with age, a combination of *SOD2* or *Nrf2* with *GSTM1* produced a statistically significant reduction in OFV (*p* < 0.05), as well. Moreover, a separation exists in the empirical Kaplan–Meier plots for the individual *SOD2* and *Nrf2* polymorphisms (Figure S2 of the Supplementary Materials), indicating that this should be further examined. However, none of the variables improved the model fit when added to the age, *GSTM1*, and *GPX1* polymorphisms. Therefore, the final hazard model for the cardiovascular survival data is calculated as follows:

$$h_{t}=\frac{1.64}{Te}\cdot {\left(\frac{t}{Te}\right)}^{0.64},\;\mathrm{where}\;Te=169\cdot {\left(\frac{AGE}{65}\right)}^{-2.61}\cdot \{\begin{array}{c} e^{0.849},\;ifGPX1\;Leu/Leu\;genotype\\ e^{-0.632},\;if\;GSTM1\;null\;genotype \end{array}$$

The estimated parameters and standard errors of the corresponding parameters of the final models are reported in Table S4 of the Supplementary Materials.

**Figure 2:**
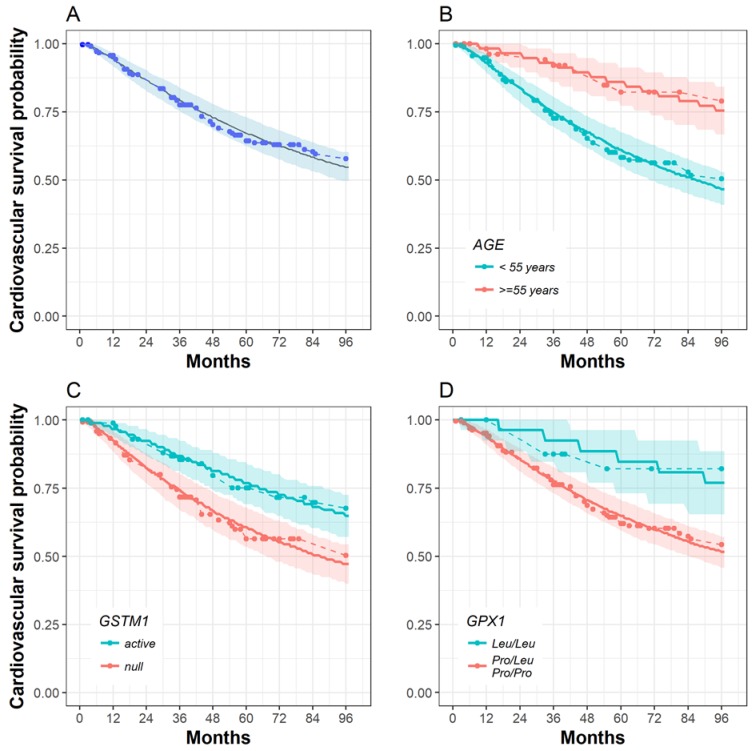
Kaplan–Meier curves for the cardiovascular survival of observed events (dashed line), including censored data (circle), and the survival model with Weibull hazard estimates (solid line) and a 90% prediction interval (shaded area). No covariate stratification (**A**); stratification by the final survival model covariates: age (**B**); the *GSTM1* active genotype is associated with longer cardiovascular survival (**C**); the *GPX1* genotype *(Leu/Leu)* genotype contributes to longer cardiovascular survival (**D**).

Good concordance was achieved between the empirical results and the median of the model-predicted Kaplan–Meier curves. The empirical Kaplan–Meier curve lies within the simulated prediction interval, indicating that there is no model misspecification (Figure 2).

In Table 5 the calculated survival probabilities for 40, 55, and 70 year-old patients, and the combination of *GSTM1* and *GPX1* polymorphisms at 3, 5, and 8 years, are presented. This table clearly indicates that age, the *GSTM1 null* genotype, and the *GPX1 Pro/Leu* or *Pro/Pro* genotypes are the main determinants of shorter cardiovascular-specific survival. The empirical Kaplan–Meier plots for the *SOD2, Nrf2*, and *GPX1* polymorphisms, which were not included in the model, are presented in Figure S1 of the Supplementary Materials.

## 3. Discussion

The results obtained in this study showed that the *SOD2 Val*/*Val* genotype is an independent risk factor for ESRD development. The risk of ESRD development increased further when the *GPX1 Leu/Leu* genotype was added into the model, although the *GPX1* polymorphism itself did not influence ESRD development. In relation to the level of oxidative stress, only the *SOD2* polymorphism was implicated as significant. On the other hand, polymorphisms in *Nrf2* and *GPX1* demonstrated an association with ESRD patients’ survival. In addition to previously established determinants of survival in ESRD patients, such as *GSTM1* polymorphism and age, the overall survival in ESRD patients was further dependent on the combination of the most favorable survival genotypes, *Nrf2 (C/C)* and *GPX1 (Leu/Leu)*. In terms of cardiovascular-specific survival, the *GPX1 (Leu*/*Leu)* genotype appeared to be a key determinant out of the three polymorphisms investigated, in combination with younger age and *GSTM1 active* genotype. Although the *SOD2* and *GPX1* polymorphisms were previously implicated in ESRD development and CKD progression [10,11,15,16], and *Nrf-2* in the cardiovascular survival of ESRD patients [7], individually, this is the first study that investigates a combination of these three polymorphisms in ESRD development and the overall or cardiovascular survival with a follow-up of up to 8 years, as well as their association with markers of protein and lipid oxidative damage byproducts, as a possible mechanism. Our results suggest that the *SOD2* polymorphism has a role in ESRD development through an oxidative stress mechanism, whereas the influence on ESRD patient survival is associated with *Nrf2* and *GPX1* polymorphisms, which are independent of the levels of oxidative stress but linked to the *GSTM1* genotype.

The published literature suggests that different causes of ESRD converge at oxidative stress as the final common pathway of renal function loss. Therefore, the research has focused on investigating the impact of functional variations of genes encoding the enzymes involved in primary antioxidant defense, such as SOD2 and GPX1 [11], on susceptibility towards ESRD. Our findings of increased ESRD risk in variant *SOD2 Val/Val* carriers is aligned with several studies concluding that this polymorphism increases the risk of diabetic nephropathy in both T1DM and T2DM patients [9,10,15]. Furthermore, Crawford et al. demonstrated that patients carrying the *Val* allele had a more rapid decline in eGFR, suggesting a faster progression of CKD in these patients [11]. Our findings, along with the aforementioned results of other investigators, support the hypothesis that a decrease in SOD2 availability contributes to the risk of ESRD. More precisely, the *Val* allele of the *SOD2* rs4880 gene results in reduced mRNA expression and production of unstable mRNA. This leads to the impaired import of the SOD enzyme into mitochondria and consequently lowers the antioxidant defense mechanism [17]. Furthermore, our findings in relation to other major antioxidant enzyme polymorphisms, such as *GPX1,* are also in concordance with a previously published study, which shows no link between the *GPX1* polymorphism and CKD development [16], although in our study we demonstrated a greater than 3-fold increased risk of ESRD when the *SOD2 Val/Val* and *GPX1 Leu/Leu* polymorphisms were combined together. Furthermore, the *Nrf2* polymorphism was investigated in our study for the first time in terms of the risk of ESRD development and the overall survival of ESRD patients, which showed an important role in ESRD survival and no influence of ESRD development.

Since oxidative stress biomarkers are significantly elevated in CKD patients, oxidative stress is emerging as an important contributing factor of CKD pathology and progression [13,14]. Nevertheless, we are the first group to investigate whether the polymorphisms of enzymes involved in the primary defense against the reactive oxygen species (ROS), *SOD2* and *GPX1*, can influence an individual’s susceptibility towards increased levels of a panel of oxidative stress biomarkers. Our findings indicate that only the *SOD2* polymorphism significantly correlates with the estimated biomarkers of oxidative stress. Regarding the byproducts of protein oxidative damage, we showed statistically significant differences only in terms of thiol groups, although other investigated biomarkers of protein oxidative damage showed a trend towards an increase in low-activity, *Val*, homozygotes. Our results are in line with GENEDIAB study, where the *SOD2* rs4880 polymorphism was associated with advanced oxidation protein products (AOPP) elevation and lower SOD2 activity, among 310 diabetes mellitus patients who developed diabetic nephropathy [15]. The variant *SOD2* genotype also correlated with the byproducts of lipid oxidative damage in the plasma of ESRD patients.

Regarding the *GPX1* polymorphism, we did not show any statistically significant association with the oxidative phenotype. The only study correlating other *GPX1* gene polymorphisms (rs1987628, rs8179164, rs9819758, rs3448) and the biomarkers of oxidative stress (AOPP and isoprostanes) was reported by Mohammedi et al. [18]. Their analysis of three out of four investigated *GPX1* polymorphisms showed no association with ESRD incidence or with oxidative stress parameters among type 1 diabetes mellitus patients, which is in line with our study, although our study had a very small percentage of diabetic patients (13%).

Surprisingly, the *Nrf2* polymorphism, rs6721961 (-617C/A), located in the promotor region of the gene and associated with the attenuated binding of Nrf2 to the ARE, which results in decreased Nrf2-dependent gene transcription, did not correlate with the level of oxidative stress byproducts in ESRD. Studies conducted in rats with 5/6 nephrectomy-induced CKD have revealed that, despite the presence of oxidative stress and inflammation, a marked decline in nuclear Nrf2 was exhibited [19]. Despite recent progress in the elucidation of human Nrf2-dependent transcriptome [2,3], further studies are needed to establish a sequence of changes in the Nrf-dependent proteome in the course of the progression of kidney deterioration towards ESRD.

This study also examined the predictive value of the polymorphism of *SOD2*, *GPX1*, and *Nrf2* genes in the overall and cardiovascular survival in ESRD patients undergoing haemodialysis. Overall survival in ESRD patients was dependent on the combination of the most favorable overall survival genotypes, *Nrf2 (C/C)* and *GPX1* (*Leu*/*Leu*), whereas only the variant *GPX1 (Leu*/*Leu)* genotype contributed to the longer cardiovascular survival. Our result showing the importance of Nrf2 in the overall survival of ESRD patients should also be explored further in light of previous reports, which suggested that CKD patients would benefit from novel therapeutic agents capable of inducing Nrf2 activation, such as bardoxolone methyl [20]. Unexpectedly, in our study, the low activity *GPX1* genotype positively affected both overall and cardiovascular survival in ESRD patients. One of the possible explanations could be that more active GPX1 contributes to a further decrease of already depleted glutathione stores. Moreover, it seems that dialysis patients do not differ in terms of *GPX1* polymorphism-associated survival from the general population. Namely, Soerensen et al. found that the variant *GPX1* is also associated with better survival in the older population [21]. Although this observation is conflicting, it seems that certain level of oxidative stress might have beneficial effects, especially on cardiovascular functions. Moderate levels of ROS or a temporary increase in ROS levels via NOX4 have vasoprotective effects on endothelial or cardiomyocyte homeostasis [22]. This suggests that a balance between the beneficial and harmful effects of ROS is important in terms of cardiovascular health.

The limitations of our study are a relatively small sample size with limited number of patients <40 years (7.4%), and a low incidence of particular polymorphisms. Consequently, rather high relative standard errors for some of the estimated parameters were obtained in the final models. External validation of the models would be valuable to confirm our results but would require data from other studies or new prospective clinical studies in ESRD patients.

## 4. Conclusions

In summary, our study shows an important novel role and mechanism of the *SOD2*, *Nrf2*, and *GPX1* polymorphisms in ESRD patients in terms of both development and survival, which should be investigated further. Our panel of biomarkers, involving various combinations of *SOD2*, *Nrf2*, and *GPX1* polymorphisms, as well as *GSTM1* polymorphisms and age, could stratify Caucasian individuals who are at increased risk of ESRD development. The same multimarker panel can be explored as a predictor of ESRD patient survival, antioxidant treatment, or targeted therapies, such as Nrf-2 activation agents. A predictive precision medicine strategy relies on the development of multi-biomarker panels, which include specific combinations of biomarkers that reflect the different pathophysiological processes underlying ESRD.

## 5. Materials and Methods

### 5.1. Study Population

A total of 256 Caucasian patients maintained on haemodialysis treatment (3 times a week for at least 3 months before the study onset) and 374 controls were enrolled. Patients over 21 years of age with stable ESRD were recruited from two dialysis centers in Belgrade, Serbia (Center for the Renal Diseases, Zvezdara University Medical Center and Department of Nephrology and Hemodialysis, University Teaching Hospital Zemun) and one center from Lazarevac (Special Hospital for Balkan Endemic Nephropathy). Patients with a previously registered malignancy or infectious co-morbidities were excluded from this study. The distribution of the underlying ESRD aetiology was as follows: hypertensive nephrosclerosis (*n* = 94), glomerulonephritis (*n* = 34), diabetic nephropathy (*n* = 24), polycystic renal disease (*n* =18), pyelonephritis (*n* = 17), Balkan endemic nephropathy (*n* = 62), obstructive nephropathy (*n* = 3), and unknown (*n* = 4). The control group was comprised of 374 individuals admitted to the same hospitals during the same period of time for routine checkup. All subjects gave their informed consent for inclusion before they participated in the study. The study was conducted in accordance with the Declaration of Helsinki, and the protocol was approved by the Ethics Committee of the Faculty of Medicine, University of Belgrade (No. 28/XII-14).

Out of the total number of study participants, 216 patients (84%) with ESRD were followed for the 8 year period (February 2010–February 2018) until the defined outcome: death or end of the follow-up period. Subjects’ data were censored if patients dropped out from the study or if they underwent kidney transplantation. Overall and cardiovascular mortality were registered 96 months from the time of the study’s onset. A cardiovascular cause of death was defined as myocardial infarction, cerebral vascular insult, heart failure, and sudden cardiac death.

### 5.2. Analysis of the SOD2, GPX1, Nrf2, and GSTM1 Genotypes

The total DNA was purified from EDTA-anticoagulated peripheral blood obtained from ESRD patients and controls, using a QIAamp DNA Blood Mini Kit (Qiagen, Germany), as per the manufacturer’s instructions.

The *GPX1* (rs1050450) polymorphism was determined by Polymerase Chain Reaction-Restriction Fragment Length Polymorphism (PCR-RFLP) [23]. To determine the *SOD2* (rs4880) polymorphism, real-time PCR (qPCR) was performed on Mastercyclereprealplex (Eppendorf, Germany), using a TaqMan Drug Metabolism Genotyping assay (Applied Biosystems, United States; ID: C_8709053_10). The analysis of the *Nrf2* polymorphism (rs6721961) was performed by the confronting two-pair primers (CTPP) method [7]. The *GSTM1* deletion polymorphism was determined by multiplex PCR, as previously described [13].

### 5.3. Oxidative Stress Byproducts Measurement in Plasma of ESRD Patients

Most oxidative stress byproducts were analyzed by spectrophotometry according to their respective methods. Protein thiol groups were analyzed according to Jocelyn’s method [24], advanced oxidation protein products (AOPP) by a modified method as per Witko-Sarsat et al. [25], MDA as per the method described by Dousset et al. [26], TOS as per Erel’s method [27], and PAB as per the method described by Alamdari et al. [28]. Furthermore, the OxiSelectTM ELISA kits (Cell Biolabs, San Diego, CA, USA) were used for determining plasma carbonyl groups, nitrotyrosine and MDA adducts, according to the manufacturer’s instructions.

### 5.4. Data Analysis

The statistical analyses were performed using Statistical Package for the Social Sciences (SPSS) ver. 17.0 (Chicago, IL, USA). Differences between the groups were compared using the χ2 test for categorical variables. In order to test the deviation of the genotype distribution, the Hardy–Weinberg equilibrium χ2 test was used. Student’s t-test, Mann-Whitney, ANOVA with Bonferroni post hoc correction, and Kruskal–Wallis tests were used to compare continuous variables, where appropriate. To assess the contribution of the genetic polymorphisms to the ESRD risk, logistic regression was performed and adjusted for confounding factors, including age and gender.

### 5.5. Time-to-Event Modelling

The exploration and visualization of data, as well as population modelling and the evaluation of the overall and cardiovascular survival (time-to-event) model, were performed on MonolixSuite (version 2018R2, France, Lixoft SAS, 2018) using the stochastic approximation expectation maximization (SAEM) algorithm and the Markov Chain Monte Carlo procedure [29]. Additionally, the R (version 3.5.1, The CRAN project) with dplyr (version 0.7.5), ggplot2 (version 2.2.1), and Rsmlx (version 1.1.0) packages were used.

Several hazard functions were tested to describe the survival curve, and the best fit hazard model was chosen based on the lowest Akaike (AIC) and Bayesian information criterion (BIC) values, as well as standard diagnostic plots. The effects of patient’s characteristics, and *SOD2*, *GPX1*, *Nrf2*, and *GSTM1* polymorphisms, as well as their combinations, were investigated in a stepwise manner. Pearson correlation analyses and the Wald test were conducted to determine significant covariates. As part of the covariate model building, we followed standard modelling procedures [30,31,32] using both the typical criterion of *p* < 0.05, as well as more stringent criterion of *p* < 0.01. Covariates were introduced sequentially in the model when they produced a statistically significant reduction (*p* < 0.05) in the objective function value (OFV). The final model was determined by backward elimination of the insignificant covariates, i.e., covariates were kept in the final model only when removing them from the full model, which resulted in an increase in the OFV at a statistical significance of *p* < 0.01. Furthermore, additional criteria for the retention of a covariate in the model included (i) a reduction in subject variability in the corresponding survival model parameter, (ii) improvement in the precision of the parameter estimates, and (iii) improvement of the goodness-of-fit plots. Moreover, biological plausibility was taken into the account as part of the covariate selection. OFV change was tested for its significance via a likelihood ratio test. The final model includes the combined influence of statistically significant covariates and an estimate of the parameters for the corresponding covariate effects. The evaluation of the model was performed using bootstrap analysis (N = 500 replicates) to determine standard errors in the parameters’ estimates. Moreover, visual predictive checks (VPCs) were performed on 1000 simulations to evaluate the adequacy of the survivor functions. VPCs enabled the comparison of the simulated Kaplan–Meier curves (derived from simulated events) with the observed Kaplan–Meier estimates. The final survivor functions were used to calculate the median probability of survival at three time points (3, 5, and 8 years) for different ESRD patient populations.

## Figures and Tables

**Table 1:** Demographic and clinical characteristics of end-stage renal disease (ESRD) patients and controls.

Variable	Controls	Patients	*p*
Age (years)	61.09 ± 10.78	62.41 ± 11.91	0.155
Gender, *n* (%)			
Male	199 (53)	147 (57)	
Female	175 (47)	109 (43)	0.297
Smoking, *n* (%) ^a^			
Never	189 (51)	167 (74)	
Ever	181 (49)	58 (26)	<0.001
Hypertension, *n* (%) ^a^			
No	245 (69)	42 (20)	
Yes	109 (31)	171 (80)	<0.001
Diabetes, *n* (%) ^a^			
No	281 (100)	188 (87)	
Yes	0 (0)	27 (13)	<0.001
BMI (kg/m^2^) ^a^	26.17 ± 4.27	24.57 ± 4.07	<0.001
Biochemical serum parameters ^a^			
Urea (mmol/L)	5.36 ± 1.99	23.92 ± 5.01	<0.001
Creatinine (µmol/L)	82.09 ± 15.04	856.93 ± 233.63	<0.001
Albumin (g/L)	43.93 ± 3.79	38.61 ± 4.40	<0.001
Total cholesterol (mmol/L)	4.34 ± 0.99	4.63 ± 1.14	<0.003
TAG (mmol/L)	1.61 ± 0.59	2.06 ± 1.34	<0.001
Haemoglobin (g/L)	142.14 ± 17.23	105.17 ± 14.76	<0.001
Haematocrit (%)	41.00 ± 6.36	31.55 ± 4.56	<0.001
Serum iron (µmol/L)	19.00 ± 4.32	11.29 ± 5.97	<0.001
Ferritin (ng/mL)	56.44 ± 28.26	377.15 ± 258.81	<0.001

All results are presented as mean ± SD or percentage. ^a^ Based on the data available.

**Table 2:** Association of individual *SOD2, GPX1*, and *Nrf2* genotypes, as well as combined *SOD2/GPX1, SOD2/Nrf2*, and *GPX1/Nrf2* genotypes with a risk of ESRD.

Genotypes	Controls, *n* (%)	Patients, *n* (%)	OR (95% CI)	*p*
*SOD2* rs4880				
*Ala*/*Ala*	113 (32)	56 (23)	1.0 ^a^	
*Ala*/*Val*	167 (46)	111 (45)	1.31 (0.88–1.97)	0.180
*Val*/*Val*	79 (22)	77 (32)	2.01 (1.28–3.16)	0.002
*GPX1* rs1050450				
*Pro*/*Pro*	158 (42)	101 (40)	1.0 ^a^	
*Pro*/*Leu*	164 (45)	122 (48)	1.22 (0.86–1.72)	0.271
*Leu*/*Leu*	43 (12)	32 (12)	1.17 (0.69–1.98)	0.558
*Nrf*2 rs6721961				
*C*/*C*	241 (71)	185 (73)	1.0 ^a^	
*C*/*A*	94 (27)	64 (25)	0.87 (0.59–1.26)	0.461
*A/A*	7 (2)	4 (2)	0.75 (0.21–2.61)	0.649
*SOD2* and *GPX1*				
*(Ala*/*Ala*+*Alal*/*Val)*/(*Pro*/*Pro+Pro*/*Leu)*	230 (68)	151 (62)	1.0 ^a^	
*(Ala*/*Ala*+*Ala/Val)*/(*Leu*/*Leu)*	36 (10)	16 (7)	0.69 (0.37–1.28)	0.239
*(Val/Val)*/(*Pro*/*Pro+Pro*/*Leu)*	67 (20)	63 (26)	1.49 (0.99–2.24)	0.051
*(Val/Val)*/(*Leu/Leu)*	6 (2)	13 (5)	3.27(1.12–8.25)	0.019 *
*Nrf2* and *SOD2*				
(C/C)/(*Ala*/*Ala* + *Ala/Val)*	183 (55)	122 (50)	1.0 ^a^	
(C/C)/(*Val*/*Val)*	49 (15)	56 (23)	1.80 (1.14–2.82)	0.011 *
(C/A+A/A)/(*Ala*/*Ala* + *Ala/Val)*	76 (23)	44 (18)	0.85 (0.55–1.32)	0.465
(C/A+A/A)/*(Val*/*Val)*	22 (7)	21 (9)	1.46 (0.76–2.80)	0.256
*Nr2* and *GPX1*				
(C/C)/(*Pro*/*Pro+Pro/Leu)*	202 (60)	158 (63)	1.0 ^a^	
(C/C)/(*Leu*/*Leu)*	34 (10)	26 (10)	0.96 (0.55–1.67)	0.876
(C/A+A/A)/*(Pro*/*Pro+Pro*/*Leu)*	94 (28)	64 (25)	0.84 (0.57–1.24)	0.391
(C/A+A/A)/*(Leu*/*Leu)*	5 (2)	4 (2)	1.04 (0.27–3.97)	0.951

Adjustments–age, gender, ^a^ Reference category, OR, odds ratio; CI, confidence interval. ESRD, end-stage renal disease; *SOD2*, superoxide dismutase; *GPX1*, glutathione peroxidase, *Nrf2*, nuclear factor (erythroid-derived 2)-like 2. For *SOD2* rs4880, genotyping was successful in 244 of 256 patients and 359 of 374 controls. For *GPX1* rs1050450, genotyping was successful in 255 of 256 patients and 365 of 374 controls. For *Nrf2* rs6721961, genotyping was successful in 253 of 256 patients and 342 of 374 controls. A Bonferroni correction was applied when two genotypes were analyzed in combination with an adjusted * *p* < 0.025.

**Table 3:** Association of *SOD2, GPX1*, and *Nrf2* polymorphisms with biomarkers of oxidative damage.

Genotypes		Protein oxidative byproducts				Lipid oxidative byproducts		TOS and PAB	
		PSH mmol/l	Carbonyls nmol/g	AOPP µmol/l	Nitrotyrosine nmol/l	MDA mmol/l	MDAadd pmol/l	TOS nmol/g	PAB mmol/l
*SOD2*	*Ala/Ala ^a^*	6.9 [5.4–8.9] 100%	2.14 ± 0.13 100%	64.3 [56.7–69.1] 100%	64.5 [46.1–91.2] 100%	2.17 ± 0.78 100%	40.28 ± 8.04 100%	18.6 [13.7–34.1] 100%	142.2 [71.8–184.4] 100%
	*Ala/Val*	6.7 [5.5–8.3] 97%	2.30 ± 0.23 107%	61.1 [46.8–74.3] 95%	59.3 [45.2–87.5] 95%	2.37 ± 0.72 109%	38.93 ± 9.42 97%	18.2 [12.8–50.8] 98%	113.9 [61.8–218.3] 80%
	*Val/Val*	6.1 [5.3–7.3] 88%	2.32 ± 0.26 * 108%	64.6 [48.1–80.8] 100%	72.3 [46.1–101.2] 112%	2.57 ± 0.79 * 118%	43.17 ± 10.15 107%	24.5 [12.8–56.3] 131%	160.4 [105.1–251.5] 113%
	*Ala/Ala+ Ala/Val ^a^*	6.7 [5.5–8.5] 100%	2.23 ± 0.21 100%	62.7 [48.4–73.3] 100%	60.3 [46.1–87.5] 100%	2.29 ± 0.75 100%	39.49 ± 8.84 100%	18.4 [13.3–48.3] 100%	126.6 [67.7–211.0] 100%
	*Val/Val*	6.1 [5.3–7.3] * 91%	2.32 ± 0.26 104%	64.6 [48.1–80.8] 103%	72.3 [46.1–101.2] 119%	2.57 ± 0.79 * 112%	43.17 ± 10.15 * 109%	24.5 [12.8–56.3] 133%	160.4 [105.1–251.5] * 127%
*GPX1*	*Pro/Pro ^a^*	6.1 [5.1–8.0] 100%	2.27 ± 0.23 100%	63.8 [46.5–73.7] 100%	64.5 [50.1–93.1] 100%	2.33 ± 0.79 100%	40.17 ± 7.63 100%	17.8 [14.0–41.0] 100%	153.8 [83.8–233.0] 100%
	*Pro/Leu*	6.5 [5.5–8.3] 106%	2.29 ± 0.24 101%	63.1 [49.3–74.4] 99%	64.5 [46.1–93.1] 100%	2.34 ± 0.74 100%	41.82 ± 11.00 104%	19.1 [12.8–53.2] 107%	143.0 [71.7–226.0] 93%
	*Leu/Leu*	6.6 [5.5–7.3] 108%	2.19 ± 0.19 96%	67.5 [48.2–85.1] 106%	60.2 [44.7–78.7] 93%	2.65 ± 0.81 114%	38.91 ± 8.19 97%	27.2 [16.8–47.5] 152%	128.6 [68.4–191.8] 84%
	*Pro/Pro+Pro/Leu*	6.3 [5.4–8.1] 100%	2.28 ± 0.23 100%	63.5 [48.6–74.3] 100%	64.5 [46.1–93.1] 100%	2.33 ± 0.77 100%	41.04 ± 9.54 100%	18.2 [13.2–48.4] 100%	146.8 [78.2–229.9] 100%
	*Leu*/*Leu*	6.6 [5.5–7.3] 105%	2.19 ± 0.19 96%	67.5 [48.2–85.1] 106%	60.3 [44.7–78.7] 93%	2.65 ± 0.81 113%	38.91 ± 8.19 97%	27.2 [16.8–47.5] 145%	128.6 [68.4–191.8] 88%
*NRF2*	*C/C ^a^*	6.3 [5.5–8.0] 100%	2.24 ± 0.22 100%	65.7 [48.8–75.7] 100%	64.5 [50.1–93.1] 100%	2.39 ± 0.78 100%	40.17 ± 9.12 100%	22.0 [13.3–53.6] 100%	142.6 [83.0–216.9] 100%
	*C/A+A/A*	6.4 [5.5–8.2] 102%	2.34 ± 0.25 104%	56.7 [44.6–70.5] 86%	52.3 [38.5–87.5] * 81%	2.33 ± 0.75 97%	41.88 ± 9.83 104%	16.2 [13.2–28.3] 73%	159.4 [72.8–252.6] 112%

All values are presented as mean ± SD or median with interquartile range (IQR). ^a^ referent genotype. PSH, proteinthiol groups; AOPP, advanced oxidation protein products; MDA, malondialdehyde; MDAadd, MDA adducts; TOS, total oxidant status; PAB, prooxidant–antioxidant balance. * *p* < 0.05 when compared to the referent genotype.

**Table 4:** Model-based predicted overall survival probability.

Patient’s Characteristics			Probability (%) to Survive at Least		
Age (years)	*GSTM1* Genotype	*Nrf2+GPX1* Genotype	3 Years	5 Years	8 Years
40	*active*	*C*/*C + Leu*/*Leu*	95.91	93.10	88.73
		*C/A or A/A + Pro/Leu or Pro*/*Pro*	91.02	84.59	74.25
	*null*	*C/C + Leu/Leu*	94.11	90.00	83.52
		*C*/*A or A*/*A + Pro/Leu or Pro/Pro*	86.92	77.28	61.40
55	*active*	*C*/*C + Leu*/*Leu*	91.40	85.27	75.43
		*C*/*A or A*/*A + Pro*/*Leu or Pro*/*Pro*	80.62	65.75	40.80
	*null*	*C*/*C + Leu*/*Leu*	87.49	78.31	63.23
		*C*/*A or A*/*A + Pro*/*Leu or Pro*/*Pro*	71.23	48.16	12.48
70	*active*	*C/C + Leu/Leu*	84.68	73.22	54.14
		*C*/*A or A*/*A + Pro*/*Leu or Pro/Pro*	64.29	35.21	1.14
	*null*	*C*/*C + Leu*/*Leu*	77.42	59.79	30.42
		*C*/*A or A*/*A + Pro*/*Leu or Pro*/*Pro*	45.88	6.71	≈0

**Table 5:** Model-based predicted cardiovascular survival probability.

Patient’s Characteristics			Probability (%) to Survive at Least		
Age (years)	*GSTM1* Genotype	*GPX1* Genotype	3 Years	5 Years	8 Years
40	*active*	*Leu*/*Leu*	99.60	99.07	98.00
		*Pro*/*Leu* *Pro*/*Pro*	98.39	96.31	92.20
	*null*	*Leu*/*Leu*	98.87	97.40	94.47
		*Pro*/*Leu* *Pro*/*Pro*	95.52	89.95	79.54
55	*active*	*Leu*/*Leu*	98.43	96.42	92.42
		*Pro*/*Leu* *Pro*/*Pro*	93.85	86.35	72.81
	*null*	*Leu*/*Leu*	95.65	90.23	80.07
		*Pro*/*Leu* *Pro*/*Pro*	83.60	66.11	40.88
70	*active*	*Leu*/*Leu*	95.67	90.27	80.14
		*Pro*/*Leu* *Pro*/*Pro*	83.67	66.22	41.03
	*null*	*Leu*/*Leu*	88.26	74.92	53.58
		*Pro*/*Leu* *Pro*/*Pro*	60.49	31.29	8.11

## Supplementary Materials

The following are available online at https://www.mdpi.com/2072-6651/11/7/431/s1, Table S1: Summary of covariate testing in overall survival model building, Table S2: Final population parameter values for the overall survival model, Table S3: Summary of covariate testing in cardiovascular survival model building, Table S4: Final population parameter values for the cardiovascular survival model, Figure S1: Empirical Kaplan-Meier curve (lines) and censored data (circle) for overall survival data based SOD2 (A), Nrf2 (B) and GPX1 (C) genotype, Figure S2: Empirical Kaplan-Meier curve (lines) and censored data (circle) for cardiovascular survival data given for SOD2 (A) and Nrf2 (B) genotype.
