# Supplementary material for: Association of Nrf2, SOD2 and GPX1 Polymorphisms with Biomarkers of Oxidative Distress and Survival in End-Stage Renal Disease Patients
Source: Toxins (Basel). 2019 Jul 23;11(7):431. doi: 10.3390/toxins11070431 (PMC6669734; doi:10.3390/toxins11070431)
Supplement: Supplementary file 1 [file toxins-11-00431-s001.pdf]

Supplementary Materials: Association of *Nrf2*, *SOD2* and *GPX1* Polymorphisms with Biomarkers of Oxidative Distress and Survival in End-Stage Renal Disease Patients

Djurdja Jerotic, Marija Matic, Sonja Suvakov, Katarina Vucicevic, Tatjana Damjanovic, Ana Savic-Radojevic, Marija Pljesa-Ercegovac, Vesna Coric, Aleksandra Stefanovic, Jasmina Ivanisevic, Zorana Jelic-Ivanovic, Lana McClements, Nada Dimkovic and Tatjana Simic

Table S1. Summary of covariate testing in overall survival model building.

	p-value			
**Covariate**	**Base**	**Uni-covariate**	**Bi-coveriate**	**Three-Covariate**
Age (years)	<0.001	+	+	+
GPX1 (Pro/Pro and Pro/Leu vs Leu/Leu)	n.s.	n.s.	n.s.	n.s.
GSTM1 (null vs active)	<0.05	<0.05	+	+
Nrf2 (C/A and A/A vs C/C)	n.s.	n.s.	n.s.	n.s.
SOD2 (Val/Ala vs Val/Val)	n.s.	n.s.	n.s.	n.s.
SOD2_GPX1	n.s.	<0.05	n.s.	n.s.
SOD2_GSTM1	n.s.	n.s.	n.s.	n.s.
SOD2_Nrf2	n.s.	n.s.	n.s.	n.s.
GPX1_Nrf2	n.s.	<0.05	<0.05	+
GPX1_GSTM1	n.s.	n.s.	n.s.	n.s.
Nrf2_GSTM1	n.s.	n.s.	n.s.	n.s.

n.s. – not significant; + - covariate included in the model.

Table S2. Final population parameter values for the overall survival model.

**Parameter**	**Estimated Value**	**Standard Error**
T_{pop}	139	17.8
Age (years) effect on T	-2.27	0.5
GSTM1 (*null*) effect on T	-0.357	0.168
Nrf2+*GPX1* (*C/C*+*Leu/Leu*) effect on T	0.765	0.347
Variance of T	0.649	0.113

T_{pop}–population scale parameter indicating time at which survival equals 0.

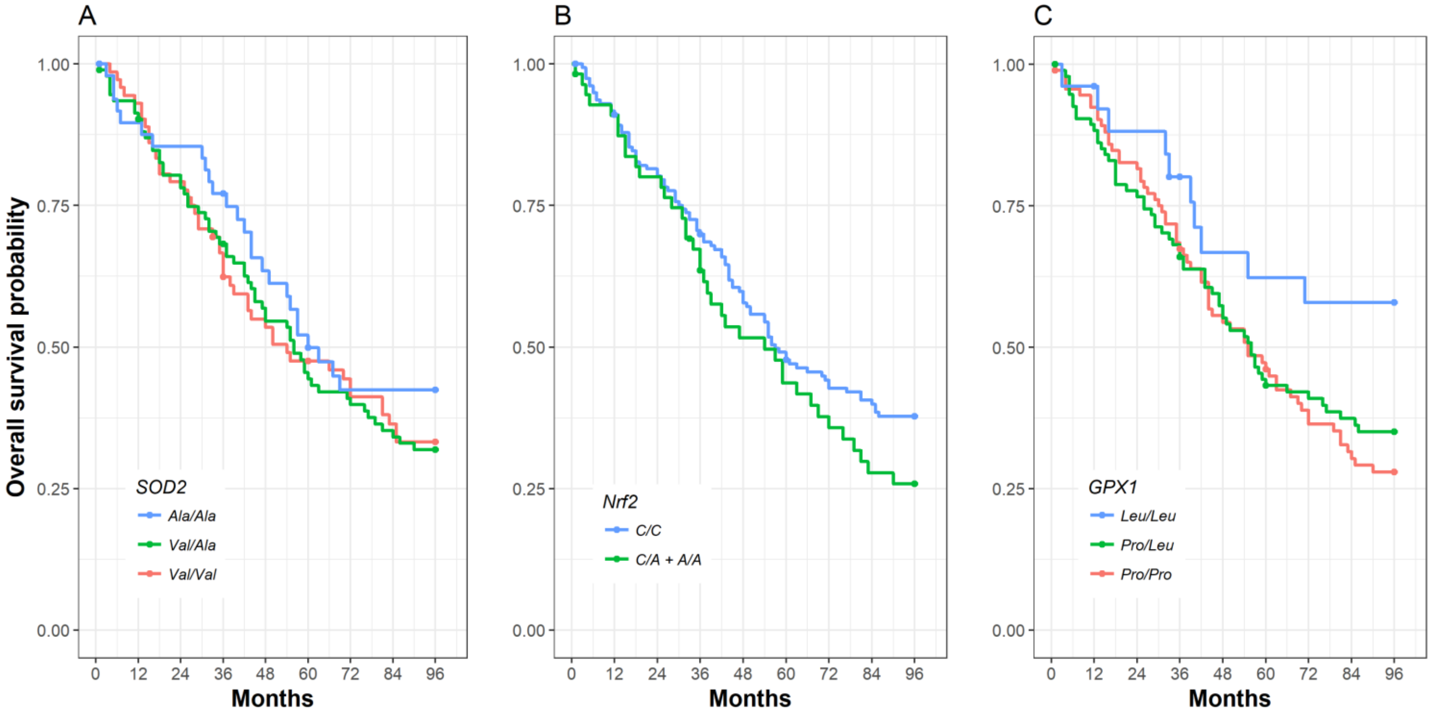

Figure S1. Empirical Kaplan-Meier curve (lines) and censored data (circle) for overall survival data based *SOD2* (**A**), *Nrf2* (**B**) and *GPX1* (**C**) genotype.

Table S3. Summary of covariate testing in cardiovascular survival model building.

	***p*-value**			
**Covariate**	**Base**	**Uni-Covariate**	**Bi-Covariate**	**Three-Covariate**
Age (years)	<0.005	+	+	+
GPX1 (Pro/Pro and *Pro/Leu vs Leu/Leu)*	n.s.	n.s.	<0.05	+
GSTM1 (active vs null)	n.s.	<0.01	+	+
Nrf2 (C/A and A/A vs C/C)	n.s.	n.s.	n.s.	n.s.
SOD2 (Val/Ala vs Val/Val)	n.s.	n.s.	n.s.	n.s.
SOD2_GPX1	n.s.	n.s.	n.s.	n.s.
SOD2_GSTM1	n.s.	<0.05	n.s.	n.s.
SOD2_Nrf2	n.s.	n.s.	n.s.	n.s.
GPX1_Nrf2	n.s.	n.s.	n.s.	n.s.
GPX1_GSTM1	n.s.	n.s.	n.s.	n.s.
Nrf2_GSTM1	n.s.	p<0.05	n.s.	n.s.

n.s.–not significant; + - covariate included in the model.

Table S4. Final population parameter values for the cardiovascular survival model.

Parameter	Estimated Value	Standard Error
T_{pop}	169	34
Age effect on T	-2.61	0.796
GPX1 (Leu/Leu) effect on T	0.849	0.457
GSTM1 (*null*) effect on T	-0.632	0.227
p_{pop}	1.64	0.357
Variance of T	0.954	0.205

T_{pop}–population scale parameter indicating time at which survival equals 0.4; p_{pop} – population shape parameter.

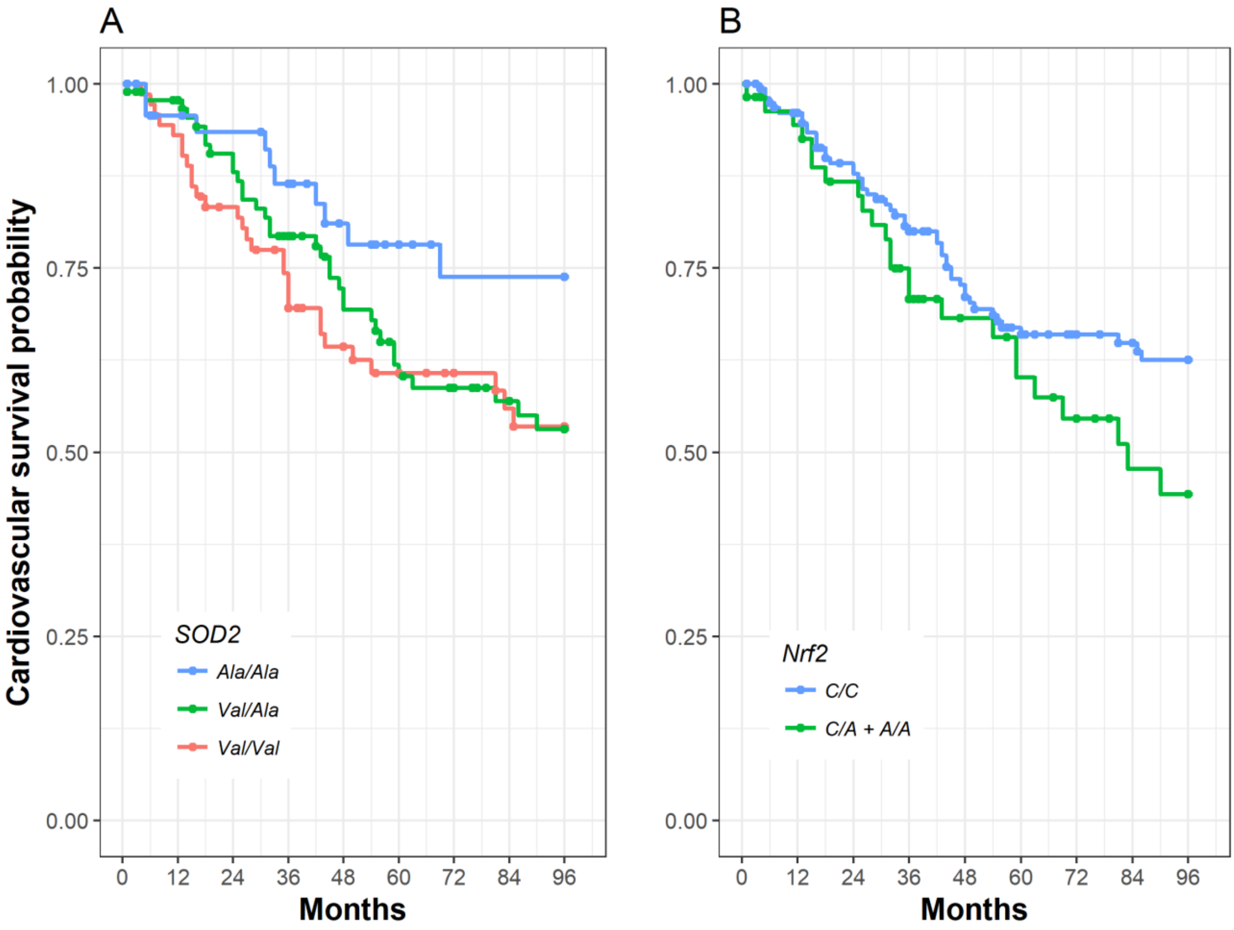

Figure S2. Empirical Kaplan-Meier curve (lines) and censored data (circle) for cardiovascular survival data given for *SOD2* (**A**) and *Nrf2* (**B**) genotype.
